# Plant‐based proteinaceous snacks: Effect of fermentation and ultrasonication on end‐product characteristics

**DOI:** 10.1002/fsn3.1705

**Published:** 2020-08-03

**Authors:** Yasaswini Kooniambedu Gunasekaran, Vita Lele, Vytaute Sakiene, Paulina Zavistanaviciute, Egle Zokaityte, Dovile Klupsaite, Vadims Bartkevics, Raquel P. F. Guiné, Elena Bartkiene

**Affiliations:** ^1^ Lithuanian University of Health Sciences Kaunas Lithuania; ^2^ Centre of Food Chemistry University of Latvia Riga Latvia; ^3^ Institute of Food Safety Animal Health and Environment BIOR Riga Latvia; ^4^ Departamento de Indústrias Alimentares, Quinta da Alagoa, Estrada de Nelas Centro de Estudos em Recursos Naturais e Ambiente, Instituto Politécnico de Viseu Ranhados Viseu Portugal

**Keywords:** biogenic amines, lactic acid fermentation, peas, ultrasound

## Abstract

The study aimed at the development of a sufficient technology to improve sensory, textural, physical, and microbiological properties of peas snacks (Ps) using solid‐state fermentation (SSF) and submerged fermentation (SMF) with two different lactic acid bacteria (LAB) strains (*Lactobacillus casei* LUHS210 and *Lactobacillus uvarum* LUHS245) for 24 hr and ultrasonication (10, 20, and 30 min). To ensure safety of the used technologies, microbiological characteristics and biogenic amines (BAs) content in treated Ps were analyzed. Additionally, a different salt content (3.6 and 1.0 g/100 g) was used for snacks preparation. The obtained results revealed that used treatments reduced enterobacteria in Ps, while in fermented Ps, yeast/moulds were not found. Ps with the lower salt content were more acidic and harder (0.90 mJ), and there was a significant effect (*p* < .05) due to the fermentation method, LAB strains, and ultrasonication on the texture of final product. Different salt content significantly affected the color coordinates of the Ps tested (*p* < .05). The predominant biogenic amines in Ps were phenylethylamine and spermidine. However, the reduction of some BAs after samples fermentation was observed. To conclude, acceptable formulations of Ps can be obtained with 1.0 g/100 g salt, and by using fermentation, as the end‐product is more attractive to consumers than those prepared with 3.6 g/100 g salt and using ultrasonication.

## Graphical abstract

Solid‐state and submerged fermentation with two different lactic acid bacteria strains (*Lactobacillus casei* LUHS210 and *Lactobacillus uvarum* LUHS245) and ultrasonication were applied for the development of a sufficient technology to improve sensory, textural, physical, and microbiological properties of plant‐based peas snacks. To ensure safety of the used technologies, microbiological characteristics and biogenic amines content in pea snacks were analyzed. Acceptable formulations of pea snacks were obtained with 1.0 g/100 g salt and by using fermentation, as the end‐product was more attractive to consumers than those prepared with 3.6 g/100 g salt and using ultrasonication.

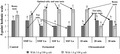

## 1 INTRODUCTION

In most European countries, current protein production is focused on proteins sourced from dairy and meat. However, there are open discussions in society about the sustainability of animal proteins and possibilities to include more plant protein sources in traditional or new food formulations. The water and nitrogen footprints of plant production are many times less than those of livestock farming (Sutton et al., 2018). For this reason, plant protein becomes a very promising environmentally friendly alternative to animal food ingredients. Some of the crops that can be used as protein sources for human consumption include beans, canola, hemp, linseed, white maize, oats, peas (Ps), potato, walnut, alfalfa (lucerne), barley, wheat, etc. (Adenekan, Fadimu, Odunmbaku, & Oke, 2018). Pea (*Pisum sativum* L.) protein is characterized as showing low allergenicity and high nutritional value and availability, as well as low cost (Shevkani, Singh, Kaur, & Rana, 2015; Stone, Karalash, Tyler, Warkentin, & Nickerson, 2015). The main challenges in utilizing Ps as a food ingredient are poor functionality and sensory characteristics unacceptable to consumers (Lam, Can Karaca, Tyler, & Nickerson, 2018). The lower consumer acceptance is mostly related with undesirable flavor compounds in peas, which are partly inherent to Ps themselves and partly developed during harvesting, processing, and storage (Roland, Pouvreau, Curran, van de Velde, & de Kok, 2017). However, Ps is rich in protein and carbohydrate, is low in fat, and contains a number of important vitamins and minerals (Zając, Klimek‐Kopyra, Mańkowski, Oleksy, & Micek, 2019). The development of new technologies for Ps treatment can be attractive for producers, as well as for consumers. Our previous studies showed that the biological value and functionality of proteinaceous plants can be increased using the antimicrobial properties shown by lactic acid bacteria (LAB) strains. Also, fermentation technology can be performed in a more sustainable manner in solid‐state fermentation (SSF) conditions (Bartkiene et al., 2019). Fermented foods may confer several benefits to human health and play an important role in a healthy and balanced diet (Nkhata, Ayua, Kamau, & Shingiro, 2018). Usually, fermentation with selected LAB leads to a reduction of undesirable microorganisms causing food spoilage; however, process conditions should be controlled, as fermentation of proteinaceous substrate can lead to the formation of biogenic amines (BAs). BAs are defined as harmful compounds produced by bacterial decarboxylation of amino acids in various foods (Mah, Park, Jin, Lee, & Hwang, 2019). However, it should be mentioned that BAs are natural endogenous compounds of plants, and their concentration depends on plant variety, maturity, and cultivation conditions (Gardini, Özogul, Suzzi, Tabanelli, & Özogul, 2016). There is legislation regarding the histamine content in fish and fish products in the USA, EU, and other countries. In contrast, the significance of BAs in fermented plant foods has been overlooked despite the presence not only of abundant precursor amino acids of BAs in plant‐based substrates, but also of microorganisms capable of producing BAs during fermentation. The toxicity of BAs can be mutually enhanced (the toxicity of tyramine and histamine can be increased by the presence of putrescine and cadaverine) (Fernández‐Reina, Urdiales, & Sánchez‐Jiménez, 2018). For this reason, the control of BA formation in new developed products is very important.

Another promising technology for proteinaceous food preparation is treatment with ultrasound. Ultrasound can be used in a diverse range of food processing applications, which are categorized according to the main mechanism by which ultrasound is effective in the process (Leong, Manickam, Martin, Li, & Ashokkumar, 2018). Maintaining food safety and preventing food spoilage require the effective destruction of pathogenic bacteria and other microorganisms (Thoma, Ellsworth, & Yan, 2018). Conventionally, thermal treatment is used to pasteurize and/or sterilize foods to make them safe for human consumption. However, thermal treatment leads to undesirable loss of vitamins, nutrients, and flavors (Lyng, McKenna, & Arroyo, 2010). Ultrasound can be used as a processing aid to inactivate microbes for the purpose of preserving food. However, ultrasonication can lead to changes of protein and other compounds in food matrices, which can be desirable (reducing unacceptable flavor), as well as undesirable (BA formation). For this reason, it is very important to evaluate BA formation after ultrasonic treatment of proteinaceous mass.

To sum up, different fermentation modes and LAB strains can have a different influence on sensory properties, functionality, and microbiological safety of Ps products, as well as BA formation. From the economical point of view, if it is possible to apply SSF, then water content could be reduced, as well as vessels used for fermentation could be smaller; however, selection of LAB should be performed, as not all strains are capable to ferment substrate at the low water content. The absence of water in SSF leads to several advantages: better product recovery, lower cost, smaller fermenter size, less chance of microbiological contamination, etc. (Pandey, 2003). Moreover, ultrasonication can also have influence on nondesirable microorganism's inactivation in Ps and induce changes in protein functionality and other compounds of Ps. For this reason, it is important to perform the comparative evaluation of these processing techniques. Therefore, this study aims at the development of a sufficient technology to improve sensory, textural, physical, and microbiological properties of peas snacks (Ps) using solid‐state fermentation (SSF) and submerged fermentation (SMF) with two different LAB strains (*Lactobacillus casei* and *Lactobacillus uvarum*) and ultrasonication (10, 20, and 30 min). *Lactobacillus casei* and *L. uvarum* were chosen for this study because according to our previous studies, these LAB strains possessed strong antimicrobial activity (Bartkiene et al., 2019). To ensure safety of the used technologies, BAs content in treated Ps was analyzed. As high levels of salt in the diet are linked to high blood pressure, which can lead to stroke and coronary heart disease (Caraher & Hughes, 2019), in our study, a different salt content (3.6 and 1.0 g/100 g) was tested in order to represent normal salt and salt‐reduced conditions and to evaluate salt impact on taste perception, texture properties, and color characteristics of produced Ps snack.

## 2 MATERIALS AND METHODS

### 2.1 Materials

Peas (Ps) were obtained from the “Galinta” enterprise (Kaunas, Lithuania) in 2018. Before treatments, Ps seeds were milled (particle size 2 mm) using a laboratory mill (Braun, Germany). The LAB strains *L. casei* LUHS210 and *L. uvarum* LUHS245 strains were obtained from the Lithuanian University of Health Sciences collection (Kaunas, Lithuania). *Lactobacillus casei* and *L. uvarum* strains were stored at −80°C in a Microbank system (Pro‐Lab Diagnostics, UK) and propagated in de Man–Rogosa–Sharpe (MRS) broth (CM 0,359, Oxoid Ltd, Hampshire, UK) at 30 ± 2°C for 48 hr, before their use for submerged fermentation (SMF) and SSF of Ps.

### 2.2 Peas fermentation with LAB

Wholemeal Ps was gently mixed with salt (3.6 or 1.0 g/100 g, according to the formulation); water and a LAB strain suspension (3% dry matter of wholemeal Ps mass), containing 8.7 log10 CFU/ml, were fermented at 30 ± 2°C for 24 hr. For 100 g of wholemeal Ps, 55 and 90 ml water was used for SSF and SMF, respectively. The final moisture content of Ps for SSF and SMF was 45 and 65%, respectively. The moisture content was determined by drying the samples at 103 ± 2°C to constant weight (ICC 109/01). Unfermented Ps were analyzed as the controls.

### 2.3 Peas treatment with ultrasound

Ultrasonication at low frequency (37 kHz, 160 W) was used for the treatment of wholemeal Ps. The equipment employed was a PROCLEAN 3.0DSP model (Ulsonix, Germany). Each 500 g sample of wholemeal Ps mixed with 250 ml of water was added to plastic bag, tied up, and placed in ultrasound bath. Samples were processed for 10, 20, or 30 min at 40°C, and each experiment was performed at least twice.

### 2.4 Peas snack sample preparation

After Ps fermentation and/or treatment with ultrasound, Ps mass was gently drained and placed in nylon containers and pressed (by using a press with a 0.5 kg weight) for 12 hr at 4°C. Ps snacks were prepared using unfermented milled Ps, Ps subjected to SSF and SMF with *L. casei* or *L. uvarum* strains, and from Ps treated with ultrasound for 10, 20, or 30 min. Ps samples for analysis were collected after 24 hr of the manufacturing process.

### 2.5 Evaluation of acidity parameters

The pH of samples was measured and recorded using a pH electrode (PP‐15; Sartorius, Goettingen, Germany). Total titratable acidity (TTA) was determined for a 10‐g sample of Ps homogenized with 90 ml of distilled water and expressed as milliliters of 0.1 M NaOH required to achieve a pH of 8.2.

### 2.6 Evaluation of pea samples color characteristics, texture parameters, and overall acceptability

The color characteristics were evaluated using a CIE L*a*b* system (CromaMeter CR‐400, Konica Minolta, Japan) (McGuire, 1992).

The texture parameter hardness was evaluated using a Brookfield CT‐3 Texture Analyser (Middleboro).

Sensory analysis of Ps was carried out according to the ISO 6658:2017(ISO^6658^, ^2017^) for overall acceptability by 20‐member panel (5 men, 15 women; age range 20–30 years) from the Lithuanian University of Health Sciences community. All Ps samples were served at room temperature (20 ± 4°C) in glass plates and tested in a sequential monadic way; samples were randomized and coded. Water was provided to rinse the palate between the samples, and panelists were asked to wait for a 30‐s period to minimize carry‐over effects. The panelists evaluated the liking of each sample on a 0 (dislike very much) to 5 (like very much) hedonic scale. Moreover, they were asked to describe the taste of Ps sample in following words: salty, beany, and sour.

### 2.7 Microbiological analysis of peas samples

LAB, spore‐forming aerobic mesophilic bacteria (TBC), total number of enterobacteria (ENT), and fungi/yeast (F/Y) counts were determined in Ps samples. For the evaluation of LAB count, 10 g of sample was homogenized with 90 ml of saline (9 g/L NaCl solution). Serial dilutions of 10^−4^ to 10^−8^ with saline were used for sample preparation. Sterile MRS agar (CM0361, Oxoid Limited) of 5 mm thickness was used for bacterial growth on Petri dishes. The dishes were separately seeded with the sample suspension using surface sowing and were incubated under anaerobic conditions at 30°C for 72 hr. The number of spore‐forming aerobic mesophilic bacteria was determined on plate‐count agar (CM0325, Oxoid Limited). Fungi and yeast were determined on chloramphenicol agar (CM0549, Oxoid Limited) after incubation at 25 ± 2°C for 5 days. The number of microorganisms was counted and expressed as log10 of colony‐forming units per gram (CFU/g). All results were expressed as the mean of three determinations.

### 2.8 Evaluation of BA content in pea samples

Sample preparation and determination of BAs in Ps samples were performed according to the method of Ben‐Gigirey, Baptista de Sousa, Juan, Villa, and Barros‐Velazquez (1999). The chromatographic analyses were carried out using a Varian ProStar HPLC system (Varian Corp.) with two ProStar 210 pumps, a ProStar 410 autosampler, a ProStar 325 UV/VIS Detector, and Galaxy software (Agilent) for data processing. For the separation of amines, a Discovery ® HS C18 column (150 × 4.6 mm, 5 µm; SupelcoTM Analytical) was used. The eluents were ammonium acetate (A) and acetonitrile (B), and the elution program consisted of a gradient system with a 0.8 ml/min flow rate. The detection wavelength was set to 254 nm, the oven temperature was 40°C, and samples were injected in 20 μl aliquots. The target compounds were identified based on their retention times in comparison with their corresponding standards.

### 2.9 Statistical analysis

All analytical experiments were carried out in triplicate. In order to evaluate the influence of three different formulas and technologies (salt concentration, fermentation, and ultrasonication) and their interaction on Ps parameters, data were subjected to analysis of variance (ANOVA) and the Tukey HSD test as a post hoc test using statistical package SPSS for Windows (v.15.0, SPSS). The results were considered statistically significant at *p* < .05.

## 3 RESULTS AND DISCUSSION

### 3.1 Acidity parameters for pea samples subjected to SSF and SMF with *L. uvarum* and *L. casei*

The pH and TTA of the Ps samples are given in Table 1. Comparing samples prepared with 3.6 g/100 g salt, the lowest pH (4.43 ± 0.01) was established after 24 hr of SSF with *L. casei*. Other samples prepared with 3.6 g/100 g salt showed a pH higher by 2.2%. Comparing samples prepared with 1.0 g/100 g salt, in both cases, SSF samples showed a lower pH than SMF samples (SSF with *L. uvarum* by 7.4%, SSF with *L. casei* by 8.6%). In most cases, lower salt content led to a lower pH of the fermented Ps samples (samples with 3.6 g/100 g salt had an average pH of 4.51; samples with 1.0 g/100 g salt had an average pH of 4.37). Comparing the TTA of the samples prepared with 3.6 g/100 g salt, the highest TTA was found for samples subjected to SSF with *L. uvarum* (5.60°N). Other samples tested showed a lower TTA, by 39.3, 1.2, and 3.6% (for SMF with *L. casei*, SSF with *L. casei*, and SMF with *L. uvarum*, respectively). In all cases, lower salt content led to higher TTA: for SMF with *L. casei* by 111.8%, for SSF with *L. casei* by 50.9%, for SMF with *L. uvarum* by 29.6%, and for SSF with *L. uvarum* by 23.2%. A very strong negative correlation between pH and TTA was established (*r* = −0.9511). Omemu, Okafor, Obadina, Bankole, and Adeyeye (2018) found the same relation between the above‐mentioned parameters during fermentation of maize: pigeon pea blends. Results of the ANOVA test indicated that there is a significant effect (*p* < .05) due to the salt concentration (3.6 or 1.0 g/100 g), fermentation method (SMF or SSF) (*p* < .05), and the interaction of these factors on the pH and TTA of Ps samples (on pH *p* < .05, on TTA *p* < .05). However, the type of microorganism applied for the fermentation was not a significant factor affecting acidity parameters.

**Table 1:** Acidity parameters (pH and total titratable acidity) of Ps prepared with 3.6 and 1.0 g/100 g salt from unfermented and fermented (in submerged and solid‐state fermentation conditions) Ps

Ps samples	pH		TTA, °*N*	
	0 hr	24 hr	0 hr	24 hr
PSLcSMF3.6 g/100 g	6.22 ± 0.01b,c	4.54 ± 0.01e	0.40 ± 0.02c	3.40 ± 0.02a
PSLcSSF3.6 g/100 g	6.35 ± 0.01d	4.43 ± 0.01c	0.50 ± 0.01d	5.50 ± 0.03c
PSLuSMF3.6 g/100 g	6.29 ± 0.01c	4.53 ± 0.01e	0.50 ± 0.03d	5.40 ± 0.02b
PSLuSSF3.6 g/100 g	6.40 ± 0.01e	4.53 ± 0.01e	0.40 ± 0.01c	5.60 ± 0.01d
PSLcSMF1g/100 g	6.20 ± 0.02b	4.63 ± 0.08f	0.30 ± 0.02b	7.20 ± 0.40g
PSLcSSF1g/100 g	6.01 ± 0.06a	4.23 ± 0.02b	0.50 ± 0.04d	8.30 ± 0.50h
PSLuSMF1g/100 g	6.14 ± 0.05b	4.48 ± 0.03d	0.20 ± 0.01a	7.00 ± 0.40f
PSLuSSF1g/100 g	6.23 ± 0.05b,c	4.15 ± 0.02a	0.20 ± 0.01a	6.90 ± 0.30e

Note

3.6 g/100 g and 1.0 g/100 g, Ps prepared with 3.6 g/100 g and 1.0 g/100 g salt, respectively. Data are represented as means (*n* = 3) ± *SE*. a–h, mean values within a column denoted with different letters are significantly different (*p* < .05).

Abbreviations: Lc, *Lactobacillus casei*; Lu, *L. uvarum*; PS, peas; SMF, submerged fermentation; SSF, solid‐state fermentation; TTA, total titratable acidity.

The decrease of acidity is an indicator of fermentation process and can be affected by many factors, such as LAB strains, carbon source, and fermentation mode. This study showed that fermentation method had a significant influence on pH and, in most cases, lower pH values and higher TTA were obtained for the peas in SSF conditions. The practice of LAB‐based food fermentations happened accidentally in the beginning, but soon spread due to its multiple benefits including preservation, safety, nutrition, and flavor (Liu & Narbad, 2018). However, information about some of the LAB starters used for Ps fermentation is scarce. In one study, in *L. plantarum*, fermentation of Ps protein‐enriched flour over the course of 11 hr, 13.5% hydrolysis, a drop in pH, and significant changes to the protein surface, and functional properties were achieved (Çabuk, Stone, Korber, Tanaka, & Nickerson, 2018). Therefore, both strains (*L. casei* and *L. uvarum*) used in this study for Ps fermentation are suitable starters, as the pH of the samples was significantly lowered. However, a significant effect of salt content on pH was also observed. A lower salt content led to a lower pH and a higher TTA of the fermented Ps, probably due to fact that the addition of salt reduces water activity, which, together with other factors, diminishes the activity of microorganisms and the chemical processes that they perform, such as production of organic acids.

### 3.2 Color coordinates and hardness of the pea samples

The color coordinates and hardness of the Ps samples are given in Table 2. Comparing the color coordinates of the fermented samples prepared with 3.6 g/100 g salt, in all cases, fermentation increased the lightness (L*) and yellowness (b*) of the samples, compared with the control (L* and b* increased by 151.1% and 294.8%, 152.5% and 279.5%, 184.1% and 304.1%, and 181.9% and 299.5% for SMF and SSF with *L. casei* and *L. uvarum* samples, respectively). Also, higher L* and b* coordinates were established for the SSF samples, compared to SMF samples (on average 12.4% and 3.7% higher, respectively). Opposite tendencies of the samples’ redness coordinates (a*) were found; compared with the control samples, lower a* coordinates were established for the fermented samples in all cases. As well as that more green than yellow color was expressed in SSF samples (for SMF with *L. casei* and *L. uvarum*, a* coordinates were 0.38 and 0.40, respectively; for SSF with *L. casei* and *L. uvarum*, a* coordinates were −1.93 and −1.34, respectively). Different tendencies were found for the color coordinates of samples ultrasonicated for 10 and 30 min: ultrasonication increased the lightness (L*) and yellowness (b*) of the samples compared with the control (L* increased by 184.0% and 97.2%, and b* increased by 307.8% and 304.9% for samples ultrasonicated for 10 and 30 min, respectively). The same tendency was observed by Belgheisi and Esmaeil Zadeh Kenari (2019) after ultrasound treatment in apple juice. Also, for the samples ultrasonicated for 10 and 30 min, more green than yellow color was expressed (a* coordinates for samples ultrasonicated for 10 min: −0.16; 20 min: −0.84). However, opposite tendencies were found for the samples ultrasonicated for 20 min; their L* and b* color coordinates were 55.9 and 11.6% lower, compared with control samples, and more yellow than green color was expressed (a* coordinates: 0.37).

**Table 2:** Color coordinates (L*, a*, b*) and texture of Ps prepared with 3.6 and 1.0 g/100 g salt from ultrasound‐treated (10, 20, 30 min), unfermented, and fermented (in submerged and solid‐state fermentation conditions) Ps

Parameters	Control	Fermented		Ultrasonicated		10 min	20 min	30 min
		SMF		SSF				
		Lc	Lu	Lc	Lu			
With 3.6 g/100 g salt								
L*	26.2 ± 0.1c	65.9 ± 0.1e	66.2 ± 0.1e	74.5 ± 0.2e	73.9 ± 0.1e	74.5 ± 0.2e	11.6 ± 0.1c	74.9 ± 0.2e
a*	3.8 ± 0.1a	0.4 ± 0.1a	0.4 ± 0.1a	−1.9 ± 0.1a	−1.3 ± 0.1a	−0.2 ± 0.02a	0.4 ± 0.03a	−0.8 ± 0.1a
b*	8.3 ± 0.1b	32.8 ± 0.1c	31.5 ± 0.2c	33.6 ± 0.1c	33.2 ± 0.2c	33.9 ± 0.1c	7.4 ± 0.1b	33.7 ± 0.1c
Texture, mJ	0.2 ± 0.01a	0.7 ± 0.01b	1.1 ± 0.01b	0.4 ± 0.01a	1.1 ± 0.01a	0.4 ± 0.01a	0.3 ± 0.01a	0.3 ± 0.01a
With 1.0 g/100 g salt								
L*	72.5 ± 1.0e	87.8 ± 6.1f	84.8 ± 4.3f	84.1 ± 2.1f	89.8 ± 12.9f	72.8 ± 2.1e	72.4 ± 1.3e	75.7 ± 1.2e
a*	8.9 ± 0.4b	6.9 ± 2.9b	4.8 ± 1.4b	5.2 ± 0.2b	5.9 ± 0.4b	8.6 ± 0.3b	8.9 ± 1.9b	8.8 ± 0.1b
b*	49.6 ± 0.4d	43.6 ± 2.01d	44.2 ± 2.3d	45.6 ± 1.8d	38.6 ± 2.3d	48.9 ± 1.6d	49.2 ± 0.2d	52.0 ± 0.4d
Texture, mJ	0.9 ± 0.02b	0.2 ± 0.01a	0.6 ± 0.01a	0.4 ± 0.01a	1.3 ± 0.03b	0.7 ± 0.02b	0.6 ± 0.01a	0.4 ± 0.01b

Note

Data are represented as means (*n* = 3) ± *SE*. a–f, mean values denoted with different letters are significantly different (*p* < .05).

Abbreviations: a*, redness (−a* greenness); b*, yellowness (−b* blueness); L*, lightness; Lc, *Lactobacillus casei*; Lu, *L. uvarum*; SMF, submerged fermentation; SSF, solid‐state fermentation.

Comparing the color coordinates of the fermented samples prepared with 1.0 g/100 g salt, in all cases, higher L* coordinates were found for the fermented samples compared with control samples (by 21.0, 16.9, 16.0, and 23.8% for SMF and SSF with *L. casei* and *L. uvarum* samples, respectively). Opposite tendencies were established for the a* and b* coordinates; in all cases, fermented samples showed lower redness and yellowness compared with control samples. Similar color coordinates were found for the control and ultrasonicated samples, except for the L* and b* coordinates of samples treated for 30 min, which were 4.4% and 5.0% higher, compared with control samples.

Results of the ANOVA test indicated that there is a significant effect (*p* < .05) due to the salt concentration (3.6 or 1.0 g/100 g) on all the color coordinates tested, due to fermentation method (SSF or SMF) on L* and b* color coordinates, and due to ultrasonication on L*, a*, and b* color coordinates of the Ps samples. In addition to that, the interactions of salt content × fermentation method on L* (*p* = .023), salt content × LAB used for fermentation on b* (*p* ≤ .0001), salt content × ultrasonication on L* and a* (*p* ≤ .0001 and *p* = .009, respectively), and fermentation method × LAB used for fermentation on b* (*p* ≤ .0001) were significant.

Comparing the texture of the Ps samples, it was found that control samples prepared with different salt content had different textures: The samples with a lower salt content were harder (0.90 mJ) (Table 2). Comparing samples prepared with 3.6 g/100 g salt, in all cases, treated (fermented, as well as ultrasonicated) samples were harder, and the hardest samples were those subjected to SSF and SMF with *L. uvarum* (1.10 mJ). Comparing samples prepared with 1.0 g/100 g salt, most of the samples were softer than the controls, except samples subjected to SSF with *L. uvarum* (1.30 mJ). Results of the ANOVA test indicated that there is a significant effect (*p* < .05) due to the fermentation method (SSF or SMF), LAB used for fermentation, and ultrasonication, and interaction of the factors analyzed was significant for the texture of Ps samples.

The color changes of Ps can be explained by the fact that during fermentation, the substrate is acidified, and organic acids have an influence on oxidation processes which can lead to changes of color, as well as on the formation of texture. Salt content also has an influence on these changes, as proteins’ water‐holding capacity and water activity are influenced by the salt content. During ultrasound treatment, localized temperature increases caused by acoustic cavitation can be sufficient to split solvent/solute molecules that have diffused into the bubbles, forming radical species. In foods, water is usually the solvent present, resulting in the following reaction: H2O ↔ 2H + OH; that is, water molecules can be split into highly reactive hydrogen and hydroxyl radicals. Radical species can also be used to promote crosslinking of proteins to form larger networks and structures (Thoma et al., 2018). All these changes can lead to specific texture formation, as well as characteristic color changes. Food materials are not homogeneous and contain a number of different components such as moisture, plant cells, and proteins. These will interact with the applied ultrasound in different ways. For example, different components will have different absorption and compression behaviours (Leong et al., 2018). Proteins and polysaccharides in foods can be subject to aggregation, particularly by heating, which can increase the viscosity of the food materials, making them more difficult to process. Shear force induced by cavitation has been shown to be highly effective in reducing the viscosity of a range of food products (Lyng et al., 2010; Thoma et al., 2018). This is achieved by breakup of the protein aggregates formed by disrupting hydrophobic interactions or by reducing the polymer chain length. Finally, more parameters (sensory properties, BAs, biosafety, etc.) should be evaluated to identify which process is the most applicable for Ps treatment.

### 3.3 Microbiological characteristics of the pea samples

Microbiological characteristics of the Ps samples are given in Table 3. In unfermented Ps samples, LAB were not found; however, after fermentation, LAB count increased, on average, in samples prepared with 3.6 g/100 g salt in SMF conditions to 9.1 log10 CFU/g, and in SSF conditions to 8.9 log10 CFU/g, with the highest count found for SSF with *L. uvarum* (9.50 ± 0.12 log10 CFU/g). Comparing samples prepared with 3.6 g/100 g and 1.0 g/100 g salt, in all cases, a lower LAB count was established in samples prepared with 1.0 g/100 g salt; LAB count ranged from 7.20 ± 0.10 to 7.49 ± 0.13 log10 CFU/g for SMF with *L. uvarum* and *L. casei*, respectively, and from 6.90 ± 0.16 to 7.90 ± 0.16 log10 CFU/g for SSF with *L. uvarum* and *L. casei*, respectively.

**Table 3:** Microbiological characteristics of Ps prepared with 3.6 and 1.0 g/100 g salt from ultrasound‐treated (10, 20, 30 min), unfermented, and fermented (in submerged and solid‐state fermentation conditions) Ps

Parameters	Control	Fermented				Ultrasonicated		
		SMF		SSF		10 min	20 min	30 min
		Lc	Lu	Lc	Lu			
With 3.6 g/100 g salt								
ENT	3.93 ± 0.12	nd	nd	nd	nd	nd	nd	nd
LAB	nd	9.08 ± 0.21b	9.11 ± 0.10b	8.30 ± 0.13a	9.50 ± 0.12c	nd	nd	nd
TBC	nd	9.05 ± 0.20a,b	8.80 ± 0.10a	9.40 ± 0.13c	9.30 ± 0.10c	nd	nd	nd
M/Y	3.23 ± 0.10a	nd	nd	nd	nd	4.5 ± 0.10c	3.90 ± 0.12b	3.83 ± 0.11b
With 1.0 g/100 g salt								
ENT	3.28 ± 0.20	nd	nd	nd	nd	nd	nd	nd
LAB	nd	7.49 ± 0.13b	7.20 ± 0.10a	7.90 ± 0.16c	6.90 ± 0.16a	nd	nd	nd
TBC	nd	7.40 ± 0.14a	7.93 ± 0.16b	7.93 ± 0.13b	7.28 ± 0.17a	nd	nd	nd
M/Y	3.42 ± 0.10a	nd	nd	nd	nd	4.25 ± 0.15c	3.93 ± 0.16b	3.80 ± 0.19b

Note

Data are represented as means (*n* = 3) ± *SE*. a–c, mean values denoted with different letters are significantly different (*p* < .05).

Abbreviations: ENT, total number of enterobacteria; LAB, lactic acid bacteria; Lc, *Lactobacillus casei*; Lu, *L. uvarum*; M/Y, fungi/yeast; nd, not determined; SMF, submerged fermentation; SSF, solid‐state fermentation; TBC, spore‐forming aerobic mesophilic bacteria.

Fermentation and ultrasonication reduced the total number of enterobacteria in Ps samples, compared with control samples which were not treated. Also, fermentation (with both strains—* L. casei* and *L. uvarum*, and in both conditions—SSF and SMF) reduced yeast/mould count, as yeast/moulds were not found in fermented samples. However, ultrasonication did not inhibit yeast/moulds, the opposite to that observed for enterobacteria.

The results of the ANOVA test indicated a significant effect (*p* < .05) of salt concentration on LAB and spore‐forming aerobic mesophilic bacteria (TBC) count (*F*(969.395) = 10.173, *p* ≤ .0001 and *F*(796.789) = 9.175, *p* ≤ .0001, respectively). The used process (fermentation or ultrasonication) was significant factor on all the tested bacteria (on enterobacteria *F*(1,186.588) = 4.034, *p* ≤ .0001; on LAB *F*(10,164.366) = 106.662, *p* ≤ .0001; on TBC *F*(9,729.365) = 112.030, *p* ≤ .0001; on fungi/yeast (F/Y) count *F*(2,696.899) = 25.171, *p* ≤ .0001). However, in compare interaction of the analyzed factors (method × salt concentration), significant influence on the LAB and TBC count was established (*F*(149.526) = 1.569, *p* ≤ .0001 and *F*(104.578) = 1.204, *p* ≤ .0001, respectively); however, interaction was not significant on enterobacteria and TBC.

The obtained results in our study could be explained by the different mechanism of microbial inactivation by LAB and ultrasound. The antimicrobial properties of LAB are explained as complex antagonistic systems produced by the starter cultures (De Vuyst & Vandamme, 1994). The Lactobacillus group produces many antimicrobial compounds, including lactic and acetic acids, which reduce environment pH and are antagonistic to a wide spectrum of pathogenic and opportunistic microorganisms. These types of microorganisms grow and are viable at specific pH range. Organic acids, produced by LAB, lower environment pH, limit the growth of bacterial pathogens (Kim & Ndegwa, 2018). Other antimicrobial compounds produced by LAB in much smaller amounts are formic acid, free fatty acids, ammonia, ethanol, hydrogen peroxide, diacetyl, acetoin, 2,3‐butanediol, acetaldehyde, benzoate, bacteriolytic enzymes, bacteriocins, and antibiotics, as well as several less well‐defined or completely unidentified inhibitory substances (Bartkiene et al., 2019; Othman, Ariff, Rios‐Solis, & Halim, 2017). Our previous studies showed that the highest antimicrobial activity was established for *L. casei* and *L. uvarum*, as these strains inhibited all the opportunistic pathogenic strains tested (Bartkiene et al., 2019). *L. uvarum* belongs to the Lactobacillus salivarius group, which comprises 25 species, 11 of which were described in the last few years (L. aquaticus, L. cacaonum, L. capillatus, L. ceti, L. ghanensis, L. hayakitensis, L. hordei, L. oeni, L. pobuzihii, L. sucicola, and *L. uvarum*) (Salvetti, Torriani, & Felis, 2012). To date, no data on the antimicrobial properties of *L. uvarum* are available, and our study showed that this strain is very promising for food fermentation. The *Lactobacillus casei* group consists of three species (*L. casei*, *L. paracasei*, and *L. rhamnosus*). These strains are able to produce acetoin, and all of them form L(+)‐lactic acid (Salvetti et al., 2012).

The use of ultrasound for microbe deactivation is considered a nonthermal food processing technique, since deactivation occurs without requiring the heating of bulk food material to high temperatures. Although ultrasound has some effect in deactivating microorganisms when applied alone, it is most effective when applied in conjugation with other processes (heating, chemical treatment, etc.). Hashemi, Abhari, and Mousavi Khaneghah (2020) reported that treatments with lactic acid and ultrasound and their combinations significantly reduced inoculated pathogen count in fresh radish. However, it was published that ultrasonication alone cannot inactivate bacteria on chicken meat; other reports are conflicting (Lyng et al., 2010). Rather, the relative stiffness and thickness of bacterial cell walls are considered to be the most important factors. Bacteria with thin, stiff cell walls are more susceptible to critical failure when subjected to ultrasonic vibration, whereas those comprising thicker, more flexible cell wall materials can dampen the mechanical effects due to cavitation. This study showed that fermentation is a more suitable technology for improving the biosafety of Ps samples, as the ultrasound technique was not capable of reducing yeast/mould count.

### 3.4 BA concentration in pea samples

The BA concentration in Ps samples prepared with 3.6 and 1.0 g/100 g salt, from ultrasound‐treated (10, 20, 30 min), unfermented, and fermented (in SMF and SSF conditions) Ps is given in Table 4. Comparing samples prepared with 3.6 g/100 g salt, no histamine and tyramine was determined in fermented and ultrasonicated samples, and a lower concentration of phenylethylamine and putrescine was found in treated samples. The phenylethylamine concentration in fermented samples, compared with controls, was 27.7%, 44.5%, 12.4%, and 17.3% lower in samples subjected to SMF and SSF with *L. casei* and *L. uvarum*, respectively. Also, treatment with ultrasound for 10, 20, and 30 min reduced the phenylethylamine concentration in samples by 65.3%, 5.4%, and 29.8%, respectively. Similar tendencies were established for putrescine, which was reduced by 70.3%, 68.7%, 38.8%, and 20.1% in samples subjected to SMF and SSF with *L. casei* and *L. uvarum* samples, respectively. In addition to that, a concentration of putrescine lower by 63.4%, 31.7%, and 75.3% was found in samples ultrasonicated for 10, 20, and 30 min, respectively, compared with control samples. Cadaverine concentrations lower than 10 mg/kg were established in three samples: in those subjected to SSF with *L. casei* and *L. uvarum* (5.9 ± 1.2 and 7.5 ± 1.0 mg/kg, respectively), and in those treated with ultrasound for 20 min (7.8 ± 1.3 mg/kg). The spermidine concentration in control samples and for SMF with both strains was, on average, 66.7 mg/kg. Different tendencies were established for the spermidine concentration in SSF, as in SSF with *L. casei*, a concentration lower by 27.8% was found, and in SSF with *L. uvarum*, a concentration higher by 44.8% was established. Spermine was detected only in SMF samples: in SMF with *L. casei* and in SMF with *L. uvarum* (on average 2.0 mg/kg).

**Table 4:** Biogenic amine (BA) concentration in Ps prepared with 3.6 and 1.0 g/100 g salt from ultrasound‐treated (10, 20, 30 min), unfermented, and fermented (in submerged and solid‐state fermentation conditions) Ps

BAs	Control	Fermented				Ultrasonicated		
		SMF		SSF		10 min	20 min	30 min
		Lc	Lu	Lc	Lu			
With 3.6 g/100 g salt								
PHE	144.2 ± 3.2e	104.3 ± 5.6c	80.1 ± 4.6b	126.3 ± 5.8d	119.3 ± 4.7c,d	50.1 ± 2.3a	136.4 ± 5.8e	101.3 ± 6.1c
PUT	50.2 ± 2.5e	14.9 ± 1.2a	15.7 ± 2.3a	30.7 ± 3.1c	40.1 ± 2.8d	18.4 ± 2.5a,b	34.3 ± 3.7c	12.4 ± 1.8a
CAD	nd	nd	nd	5.9 ± 1.2a	7.5 ± 1.0b	nd	7.8 ± 1.3b	nd
HIS	12.0 ± 1.2	nd	nd	nd	nd	nd	nd	nd
TYR	4.1 ± 0.9	nd	nd	nd	nd	nd	nd	nd
SPRMD	66.5 ± 3.6c	67.0 ± 2.8c	66.5 ± 3.1c	48.0 ± 3.5a	96.3 ± 4.5e	78.3 ± 3.5d	52.2 ± 2.3a	58.0 ± 2.8b
SPRM	nd	2.1 ± 0.3a	2.5 ± 0.2a	nd	nd	nd	nd	nd
With 1.0 g/100 g salt								
PHE	122.21 ± 3.62c	110.89 ± 3.78b	144.02 ± 3.69d	123.73 ± 4.01c	87.81 ± 1.73a	117.50 ± 2.36c	148.82 ± 3.96d	148.58 ± 5.69d
PUT	40.83 ± 4.71a,b	35.56 ± 1.05a	44.20 ± 2.58b	47.79 ± 1.27b	39.53 ± 1.52a	37.52 ± 1.06a	41.75 ± 1.05b	44.39 ± 1.12
CAD	nd	nd	nd	nd	nd	nd	nd	nd
HIS	4.20 ± 0.21c	1.76 ± 0.19a	3.84 ± 0.25c	nd	nd	3.67 ± 0.29c	nd	2.71 ± 0.34b
TYR	nd	nd	nd	nd	nd	nd	nd	nd
SPRMD	120.99 ± 3.45b	101.50 ± 2.87a	128.46 ± 3.68c	137.22 ± 2.96c	117.03 ± 2.43b	118.56 ± 3.62b	117.97 ± 4.15b	132.66 ± 5.67c
SPRM	nd	nd	nd	nd	nd	nd	nd	nd

Note

Data are represented as means (*n* = 3) ± *SE*. a–e, mean values within a row denoted with different letters are significantly different (*p* < .05).

Abbreviations: Bas, biogenic amines; CAD, cadaverine; HIS, histamine; Lc, *Lactobacillus casei*; Lu, *L. uvarum*; nd, not determined; PHE, phenylethylamine; PUT, putrescine; SMF, submerged fermentation; SPRM, spermine; SPRMD, spermidine; SSF, solid‐state fermentation; TYR, tyramine.

Comparing samples prepared with 1.0 g/100 g salt, cadaverine, tyramine, and spermine were not found (Table 4). The predominant BAs in Ps samples prepared with 1.0 g/100 g salt were phenylethylamine, spermidine, and putrescine. Comparing phenylethylamine concentrations, the highest was found for SMF with *L. uvarum*, and for ultrasound treatment for 20 and 30 min (on average, 147.14 mg/kg), and the lowest for SSF with *L. uvarum* (28.2% lower than that in control samples). The putrescine concentration in samples ranged from 35.56 ± 1.05 to 47.79 ± 1.27 mg/kg, for SMF and SSF with *L. uvarum*, respectively. Histamine was found in five samples (control, SMF with both strains, and ultrasound treatment for 10 and 30 min), and in all cases, the content was lower than 5.0 mg/kg. The spermidine concentration in control samples was 120.99 ± 3.45 mg/kg, and comparing treated samples with controls, a higher concentration of spermidine was found for SMF with *L. uvarum*, SSF with *L. casei*, and for ultrasound treatment for 30 min (by 6.2%, 13.4%, and 9.7%, respectively). For SMF with *L. casei*, a lower concentration of spermidine was found (16.1% lower), compared with control samples; in other samples analyzed, the spermidine concentration was similar to that of the control (on average 117.9 mg/kg).

Many factors influence the production of BAs in foods, including the physicochemical parameters, pH, salt concentration, bacterial activity, humidity, storage duration, temperature, and the concentration of free amino acids (Linares et al., 2012). Different BAs biosynthesis highly depends on the activity of amino acid decarboxylases, which are widely present in most of the microorganisms; however, both biosynthesis and degradation of BAs are a species‐level characteristic in bacteria (Alvarez & Moreno‐Arribas, 2014). In our study, the reduction of some BAs after samples fermentation could be related with the fact that LAB can consume BAs precursors (i.e., amino acids), reduce the growth of spoilage microorganism, or have amine oxidase to degrade the BAs. The previous research of Bartkiene et al. (2020) showed that the total BA content in barley by‐products was reduced after SSF and SMF with P. acidilactici LUHS29. It was reported that *L. plantarum*, *L. sakei*, *L. pentosus*, Pediococcus acidilactici, and *L. casei* had the ability to degrade in vitro tyramine histamine and putrescine (Alvarez & Moreno‐Arribas, 2014). Lower water activity in SSF, ultrasound treatment, and higher salt content also prevent the growth of BA‐producing bacteria.

In our study, analyzed BAs are the most important in foods, and each of them has different precursor; for example, phenylethylamine can be formed from phenylalanine, spermidine from arginine, and the toxicity of BAs depends on synergistic effects (Suzzi & Torriani, 2015). According to our results, phenylethylamine and spermidine content in Ps was the highest, compared to other BAs. European legislation does not specify a BA threshold, but the European Food Safety Authority (EFSA) has elaborated a scientific opinion on the risk associated with the formation of BAs in fermented products (EFSA Panel on Biological Hazards (BIOHAZ), 2011). Based on the mean content in foods and consumer exposure data, fermented food categories have been ranked with respect to histamine and tyramine, but the information presently available is insufficient to conduct quantitative risk assessment of BAs, individually and in combination(s).

### 3.5 Overall acceptability of the peas snacks

**Figure 1:**
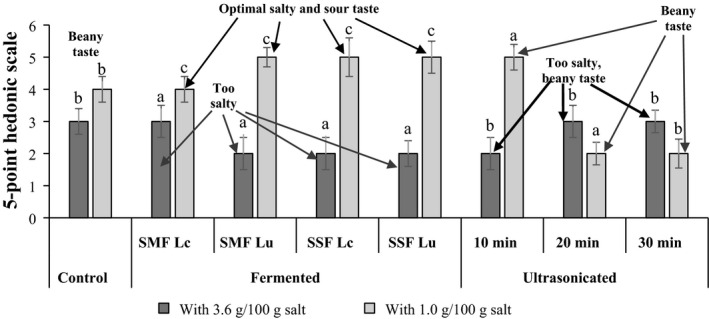
Overall acceptability of Ps prepared with 3.6 g/100 g and 1.0 g/100 g salt from ultrasound‐treated (10, 20, 30 min), unfermented, and fermented (in submerged and solid state fermentation conditions) Ps. Lc, *Lactobacillus casei*; Lu, *L. uvarum*; SMF, submerged fermentation; SSF, solid‐state fermentation. Data are represented as means (*n* = 3) ± SE. a–c, mean values denoted with different letters are significantly different (*p* ≤ .05)

Overall acceptability of the Ps samples is given in Figure 1. It was established that 3.6 g/100 g salt in the snack formula is too high, because fermented and ultrasonicated samples were evaluated by panelists as too salty. Salt is added in order to reduce the water activity, preventing the growth of undesirable microorganisms and to improve the taste as well as the texture of the final product (Pino et al., 2018). However, according to the European Commission, nutritional policies should seek to reduce salt intake (European Council, 2010). It should be mentioned that fermentation reduces the raw taste of Ps samples, and for the Ps treatment, 1.0 g/100 g salt and fermentation (SSF and SMF with both strains) can be recommended, as this formulation and technology leads to higher overall acceptability of the products (all fermented samples were evaluated by 5 points). However, treatment with ultrasound did not reduce the beany taste of the Ps; for this reason, sample acceptability was lower than for fermented samples. Usually, consumer acceptability of bean‐based products is reduced due to beany and grassy flavors (Simons & Hall, 2018). Beany and other off‐flavors of raw pulse flours are associated with volatile organic compounds such as aldehydes, ketones, and alcohols. Several techniques are used to change the flavor of pulses (Shin, Kim, & Kim, 2013). Our study showed that fermentation with *L. casei* and *L. uvarum* led to an increased acceptability of Ps. This could be associated with the release of flavorful taste and aroma compounds during degradation of proteins and carbohydrates by LAB strains and endogenous enzymes. Large amount of free amino acids and peptides are formed, which can stimulate appropriate taste receptors and change the flavor of final fermented product (Ng'ong'ola‐Manani, Østlie, Mwangwela, & Wicklund, 2014). Fermentation also increases the bioavailability of nutrients in foods (Oloyede, James, Ocheme, Chinma, & Akpa, 2016). Thus, according to this study, innovating acceptable formulations using fermented Ps can make the end‐product more attractive to consumers looking for healthier options.

## 4 CONCLUSIONS

Peas are a valuable protein source for human consumption; however, the processing of peas is complex due to poor functionality and sensory characteristics unacceptable to consumers. For this reason, different processing techniques such as ultrasonication and fermentation were applied to improve sensory, textural, physical, and microbiological properties of peas snacks. Results revealed that fermentation is a more suitable technology for ensuring the biosafety of Ps than ultrasound, as well as ensuring better acceptability of the final products. Both fermentation and ultrasonication reduced enterobacteria in Ps samples; however, in ultrasonicated samples, yeast/mould count was not reduced. Phenylethylamine and spermidine were found as predominant BAs in Ps. Finally, acceptable formulations of Ps can be obtained with 1.0 g/100 g salt and using fermentation with *L. casei* and *L. uvarum* strains, as the end‐product is more attractive than those prepared with 3.6 g/100 g salt and using ultrasonicated Ps.

## CONFLICT OF INTEREST

The authors declare that they do not have any conflict of interest.

## ETHICAL REVIEW

This study does not involve any human or animal testing.
